# Supplementary material for: The Predictive Value of Systemic Immune-Inflammation Index on Bladder Recurrence on Upper Tract Urothelial Carcinoma Outcomes after Radical Nephroureterectomy
Source: J Clin Med. 2021 Nov 12;10(22):5273. doi: 10.3390/jcm10225273 (PMC8623909; doi:10.3390/jcm10225273)
Supplement: Supplementary file 1 [file jcm-10-05273-s001.zip › jcm-1397282-supplementary.pdf]

Supplementary Materials

Table S1. Demographics and clinicopathologic characteristics of 108 patients with bladder recurrence after nephroureterectomy according to SII level.

Variables	Total	SII value		
		High	Low	*p* value
	n = 108	*n* = 70	*n* = 38	
Age (Years)				
Median (IQR)	69.5(16)	68.0(12)	70.2(17)	0.638
Gender				0.354
Male	56(51.9)	34(48.6)	22(57.9)	
Female	52(48.1)	36(51.4)	16(42.1)	
CKD stage				0.052
Stage 1	8(7.4)	2(2.9)	6(15.8)	
Stage 2	22(20.4)	14(20.0)	8(21.1)	
Stage 3	44(40.7)	28(40.0)	16(42.1)	
Stage 4	6(5.6)	6(8.6)	0(0.0)	
Stage 5	28(25.9)	20(28.6)	8(21.7)	
Bladder cancer history				**0.044**
Yes	73(67.6)	52(74.3)	21(55.3)	
No	35(32.4)	18(25.7)	17(44.7)	
Preoperative hydronephrosis				**0.018**
Yes	62(57.4)	46(65.7)	16(42.1)	
No	46(42.6)	24(34.3)	22(57.9)	
Type of operation				0.226
Open	76(70.4)	52(74.3)	24(63.2)	
Laparoscopic	32(29.6)	18(25.7)	14(36.8)	
Tumor location				0.211
Pyelocaliceal	46(42.6)	26(37.1)	20(52.6)	
Ureteral	36(33.3)	24(34.3)	12(31.6)	
Both	26(24.1)	20(28.6)	6(15.8)	
Multifocality				0.236
Single	84(77.8)	52(74.3)	32(84.2)	
Multifocal	24(22.2)	18(25.7)	6(15.8)	
Pathological T stage				0.183
pTis/pTa	70(64.8)	44(62.9)	26(68.4)	
pT1	36(33.3)	24(34.3)	12(31.6)	
pT2	2(1.9)	2(2.9)	0(0.0)	
Grade				**0.026**
High	61(56.5)	45(64.4)	16(42.1)	
Low	47(43.5)	25(35.7)	22(57.9)	

CKD = Chronic kidney disease, SII = Systemic Immure-Inflammation Index. Statistically significant values are marked with Bold.

Table S2. Collected reports of systemic inflammatory index on upper tract urothelial carcinoma.

Author	Year	Study Design	Patients	Age (years)	SII cut-off Value	Application	Outcome Measurement
Jan [22]	2019	Retrospective single center	424	68.5 ± 10.9	580	SII with MLR	OS, CSS, PFS
Zheng [23]	2020	Retrospective single center	525	67.6 ± 10.5	672	SII with PNI	OS, CSS, RFS
Mori [6]	2021	Retrospective multiple centers	2492	69	485	SII	OS, CSS, RFS
Current study	2021	Retrospective single center	376	65.9 ± 10.8	485	SII	CSS, MFS, BRFS

BRFS = bladder recurrence-free survival; CSS = cancer-specific survival; MFS = metastatic-free survival; OS = overall survival; PFS = progression-free survival; PNI = prognostic nutrition index; RFS = recurrence-free survival; SII = systemic immune-inflammation index.

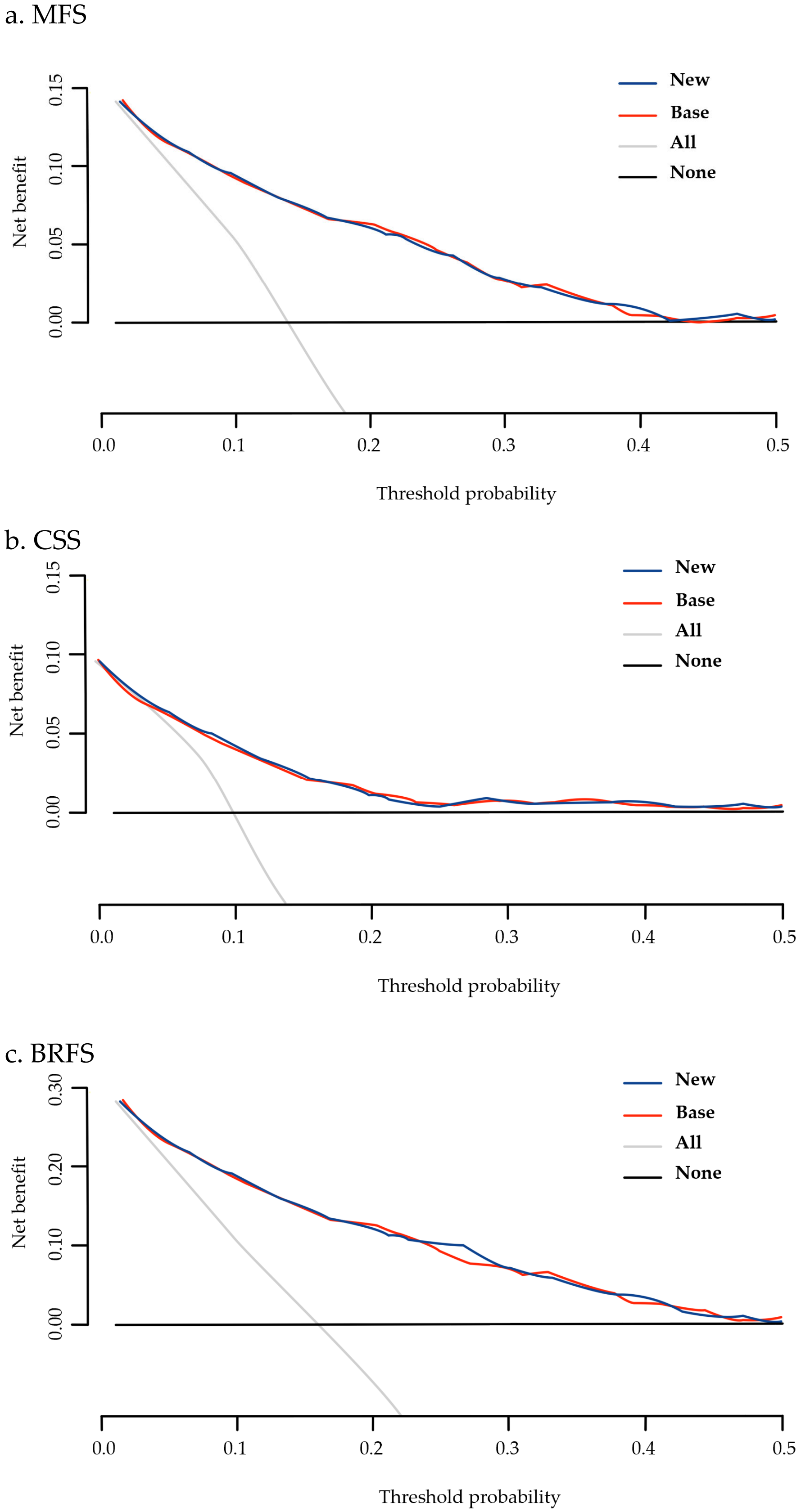

Figure S1. Decision curve analyses for net benefit of inclusion SII for prediction of survival (a) MFS, (b) CSS, and (c) BRFS.
